# Manitoba the Best

**Published:** 1894-12

**Authors:** 


### MANITOBA THE BEST.

On page 471 of this number of the JOURNAL, is a copy of the Legislation regulating veterinary practice in Manitoba. It is certainly the most distinct and definite legislation that we know of on this subject. It will be interesting to learn what provision is made for the future.

